# Transcriptome analysis reveals self-incompatibility in the tea plant (*Camellia sinensis*) might be under gametophytic control

**DOI:** 10.1186/s12864-016-2703-5

**Published:** 2016-05-17

**Authors:** Cheng-Cai Zhang, Li-Yuan Wang, Kang Wei, Li-Yun Wu, Hai-Lin Li, Fen Zhang, Hao Cheng, De-Jiang Ni

**Affiliations:** Key Laboratory of Tea Biology and Resources Utilization, Ministry of Agriculture, Tea Research Institute Chinese Academy of Agricultural Sciences, 9 Meiling South Road, Hangzhou, 310008 China; College of Horticulture and Forestry Science, Huazhong Agricultural University, No.1, Shizishan Street, Hongshan District, Wuhan, Hubei Province 430070 China

**Keywords:** *Camellia sinensis*, Theaceae, Tea, Transcriptome, Self-incompatibility, *S-RNase*, Gametophytic

## Abstract

**Background:**

Self-incompatibility (SI) is under genetic control and prevents inbreeding depression in angiosperms. SI mechanisms are quite complicated and still poorly understood in many plants. Tea (*Camellia sinensis* L.) belonging to the family of Theaceae, exhibits high levels of SI and high heterozygosity. Uncovering the molecular basis of SI of the tea plant may enhance breeding and simplify genomics research for the whole family.

**Results:**

The growth of pollen tubes following selfing and crossing was observed using fluorescence microscopy. Self-pollen tubes grew slower than cross treatments from 24 h to 72 h after pollination. RNA-seq was employed to explore the molecular mechanisms of SI and to identify SI-related genes in *C. sinensis*. Self and cross-pollinated styles were collected at 24 h, 48 h and 72 h after pollination. Six RNA-seq libraries (SP24, SP48, SP72, CP24 CP48 and CP72; SP = self-pollinated, CP = cross-pollinated) were constructed and separately sequenced. In total, 299.327 million raw reads were generated. Following assembly, 63,762 unigenes were identified, and 27,264 (42.76 %) unigenes were annotated in five public databases: NR, KOG, KEGG, Swiss-Port and GO. To identify SI-related genes, the fragments per kb per million mapped reads (FPKM) values of each unigene were evaluated. Comparisons of CP24 vs. SP24, CP48 vs. SP48 and CP72 vs. SP72 revealed differential expression of 3,182, 3,575 and 3,709 genes, respectively. Consequently, several ubiquitin-mediated proteolysis, Ca^2+^ signaling, apoptosis and defense-associated genes were obtained. The temporal expression pattern of genes following CP and SP was analyzed; 6 peroxidase, 1 polyphenol oxidase and 7 salicylic acid biosynthetic process-related genes were identified. The RNA-seq data were validated by qRT-PCR of 15 unigenes. Finally, a unigene (CL25983Contig1) with strong homology to the S-RNase was analyzed. It was mainly expressed in styles, with dramatically higher expression in self-pollinated versus cross-pollinated tissues at 24 h post-pollination.

**Conclusions:**

The present study reports the transcriptome of styles after cross- and self-pollination in tea and offers novel insights into the molecular mechanism behind SI in *C. sinensis*. We believe that this RNA-seq dataset will be useful for improvement in *C. sinensis* as well as other plants in the Theaceae family.

**Electronic supplementary material:**

The online version of this article (doi:10.1186/s12864-016-2703-5) contains supplementary material, which is available to authorized users.

## Background

Self-incompatibility (SI) is genetically controlled, and promotes outcrossing while preventing inbreeding depression in flowering plants [1]. Although SI has emerged as a model for cell-cell communication and signal transduction [2, 3], it is also an agriculturally important trait [4, 5]. Although SI has been widely demonstrated in angiosperms, its mechanism has been elucidated in only a few plants [6]. Sporophytic SI (SSI) in Brassicaceae is controlled by the stigma-specific S-receptor kinase (SRK) and pollen-specific S-locus cysteine-rich protein (SCR), which inhibits germination and growth of self-pollen on the stigma [7]. While during gametophytic SI (GSI) in *Rosaceae*, *Solanaceae* and *Plantaginaceae*, the interaction between the female determinant S-RNase and the male component S-locus F-box protein (SLF/SFB) mediate the rejection response of self-pollen tubes in the pistil [8–10]. Another GSI mechanism exists in *Papaveraceae* in which a Ca^2+^ signaling cascade leads to programmed cell death (PCD) [11]. Additionally, many species demonstrate SI from pollen inhibition in the ovary, which is called either ovarian SI (OSI) or late-acting SI (LSI). This form of SI was defined according to the location of the pollen inhibition instead of the genetic mechanism of SI as with GSI and SSI [12]. LSI is more widespread among the basal groups of Angiosperms, which indicates a conserved ancestral mechanism of SI; nevertheless, its molecular basis still remains unclear [6, 13]. Some types of LSI may be controlled by either the gametophyte or sporophyte independently, whereas other types may be jointly controlled [13].

Overall, SI is regulated by multiple genes and associated with different metabolic pathways. Next Generation Sequencing (NGS) and transcriptome analysis has revealed candidate genes that contribute to SI interaction in *Citrus clementine* [14], *Citrus limon* [4] and *Leymus chinensis* [3].

The Theaceae family consists of 20 genera and over 600 species [15]. Several of the species in these subclasses have significant economic value, such as Tea (*C. sinensis* L.), Tea-oil tree (*C. oleifera* Abel.) and the ornamental Sasanqua (*C. sasanqua* Thunb.). Due to their self-incompatibility, Theaceae species are highly heterogeneous, making crop improvement via classical breeding and genetics difficult [16], it and has hindered genetic map construction and contig assembly from whole-genome sequencing [16–18]. Therefore, uncovering the molecular basis of SI in Theaceae may simplify and enhance breeding and genomics in this family.

Tea is one of the most prominent beverages in the world [16] and is widespread in tropical and subtropical regions [15]. It is also a model for studying SI [19, 20]. Tea has exhibited GSI because of a self-pollen tube growth inhibiting at the base of the style [21, 22]. However, more recent evidence suggests LSI because the pollen tubes from self-fertilized flowers were inhibited in the ovary [23, 24]. Similarly, the edible oil plant of *C. oleifera* also manifests LSI behavior [5]. In these studies, although pollen tubes from self-pollinations penetrated the ovary, pollen tube growth was inhibited to some extent in the style. In contrast, pollen tubes from cross-pollination reached the ovary with no interference [5, 22–24]. Therefore, the differential growth of the pollen tube from self and cross-pollinations was a critical trait in understanding SI in *C. sinensis*.

Attempts have been made to understand the molecular mechanisms underlying tea SI [16, 21, 25]. A pistil-specific pathogenesis-related-1 (PR-1) protein was identified in tea, but the relationship between PR-1 and SI was not clearly demonstrated [21]. cDNA-AFLP method was also used to analyze differentially expressed genes in self-incompatible lines of tea, and 34 genes were isolated which were involved in energy metabolism, signal transduction and defense [26]. A *C. sinensis* floral transcriptome analysis revealed the presence of several pollen/pistil specific expression unigenes [16]. The objective of this study was to gain deeper insights into the molecular basis of SI and to provide a global view of candidate SI-related genes in tea using RNA-seq. We believe that the identification of candidate SI-related genes will contribute to a more complete understanding of the SI mechanism in *C. sinensis*, which will improve breeding and genomics research in the Theaceae.

## Results and discussion

### Observation of pollen tube growth

**Fig. 1:**
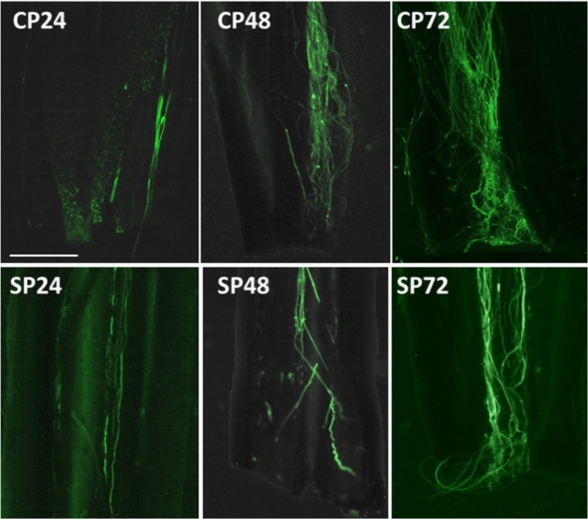
Pollen tubes growth after CP and SP in *C. sinensis*. CP24: a few pollen tubes reached the style base; CP48 and CP72: a larger number of pollen tubes arrived the style base; SP24: pollen tubes in the middle of the style; SP48: a few pollen tubes reached the style base; SP72: a large number of pollen tubes arrived the style base. Bar = 500 μm

Pollens from both the cross- and self-pollination germinated at 6 h post-pollination, and no visible differences were observed in pollen tube growth from 6 h to 12 h. At 24 h after pollination, a small number of tubes from the cross-pollination reached the style base (Fig. 1). However, pollen tubes from self-pollination grew more slowly, reaching just the middle of the style. At 48 h after pollination, most tubes from cross-pollination reached the style base, but the pollen tubes from the self-pollination took 72 h to reach the same place. Self-pollen tubes grew slower than cross-pollen tubes from 24 h to 72 h after pollination. This is consistent with previous studies that also showed reduced pollen tube growth from a SI cross [5, 21–23]. The delay in pollen tube growth may be critical for the SI reaction because, by the time that the self-pollen tubes penetrate the ovary, the pistil may have already been primed for abscission [6].

### RNA-seq and transcriptome assembly

To uncover the mechanism behind SI reaction in *C. sinensis*, six RNA-seq libraries (CP24, CP48, CP72, SP24, SP48 and SP72) were separately constructed and sequenced. In total, 299.327 million raw reads with a Q30 over 83 % were generated. After removing the low quality sequences (length < 35 bp, Q < 20), a total of 294.144 million clean reads were retained (Table 1). After *de novo* assembly using Trinity software, 63,762 unigenes were identified. The lengths of unigenes ranged from 301 bp to 14,580 bp, with an average of 1,018.26 bp and a N50 length of 1,354 bp (Table 2).

**Table 1:** Summary for the RNA-seq outcomes of six separately pooled samples

Sample	CP24^a^	CP48	CP72	SP24	SP48	SP72	Total
Raw reads (million)	43.798	55.519	54.843	48.094	49.118	47.956	299.327
Clean reads (million)	43.075	54.536	53.905	47.337	48.283	47.008	294.144
Total base (Gb)	5.007	6.339	6.266	5.502	5.612	5.464	34.189
Q30	83.3 %	83.4 %	83.5 %	83.3 %	83.1 %	83.1 %	—

^a^CP = cross pollination, SP = self pollination, numbers after CP and SP indicate hours after pollination

**Table 2:** Summary for the *de novo* assembly

	300–499 (bp)	500–999 (bp)	≥1000 (bp)	N50 (bp)	Total Length (Mbp)	Max Length (bp)	Min Length (bp)	Average Length (bp)
Unigene	19228	24178	20356	1354	61.918	14580	301	1018.26

### Functional annotation

**Fig. 2:**
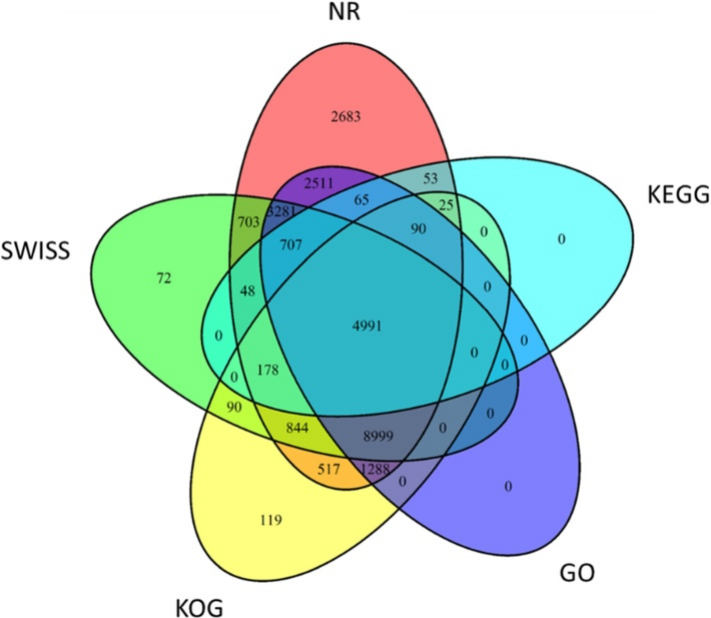
Blastx results of the transcriptome to five databases

To predict potential functions of the assembled unigenes, all of them were blastx (E-value ≤ 10^−5^) against 5 public databases, including the National Center for Biotechnology Information (NCBI) Non-redundant Protein (NR), Clusters of Orthologous Groups for Eukaryotic Complete Genomes (KOG), Kyoto Encyclopedia of Genes and Genomes (KEGG), Swiss-Prot Protein Database (Swiss-Port) and Gene Ontology (GO) (Fig. 2). Most unigenes (26,984, 42.32 %) were annotated to the NR database, and 16,210 (60.08 %) sequences had significant homology (blastx E-value < 10^−45^). Among the annotated unigenes, 37 % (9975) and 9.76 % (2631) had strong homology to *Vitis vinifera* and *Theobroma cacao,* respectively (Additional file 1).

**Fig. 3:**
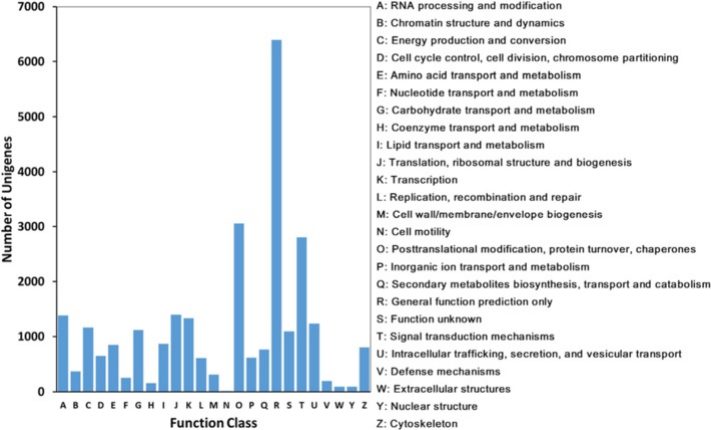
KOG functional classification of the *C. sinensis* transcriptome

The unigenes were further annotated and classified using the KOG database (Fig. 3). A total of 27,618 unigenes were assigned KOG classifications. Among the 25 KOG categories, the “general function prediction only” (6391, 23.15 %) was the most abundant, followed by “posttranslational modification, protein turnover, chaperones” (3054, 11.06 %), “signal transduction mechanisms” (2,803, 10.15 %) and “translation, ribosomal structure and biogenesis” (1394, 5.05 %).

The unigenes were then matched to the GO terms (Additional file 2). A total of 21,932 unigenes were classified into 58 sub-categories belonging to 3 categories (biological process, cellular component and molecular function). Among the biological process category, “metabolic process” and “cellular process” were the main functional groups, which were followed by “single organism process” and “response to stimulus”. In terms of cellular component, “cell part” and “cell” were the most highly represented subcategories. For the molecular function category, “binding” and “catalytic activity” were the two main groups.

To predict putative functions of the unigenes, KEGG annotation was also performed. Altogether, 6158 unigenes were assigned to 330 pathways, including “plant hormone signal transduction”, “calcium signaling pathway”, “plant-pathogen interaction”, “flavonoid biosynthesis” and “ubiquitin mediated proteolysis”. Plant hormones play an important role in incompatibility response; for example, in *Theobroma cacao*, auxin exhibited a strong increase after compatibility pollination, while ethylene exhibited a strong increase after incompatibility pollination [27], and higher 1-naphthaleneacetic acid (NAA) concentrations improved flower retention after incompatibility pollination [28]. Plant hormone signal transduction-related pathways were enriched in tomatoes when comparing the unpollinated and pollinated styles of self-compatible and self-incompatible species [29]. Calcium is essential to pollen tube germination and growth, and its temporal and spatial changes in cytosol generate Ca^2+^ signals [30, 31]. Ca^2+^ signaling cascade leads to PCD, which mediates SI reaction in *Papaveraceae* [11]. RNA-seq has also revealed that the calcium-signaling pathway is associated with SI in *Citrus* [14, 32] and *Leymus chinensis* [3]. SI systems presumably have evolved from pathogen defense mechanisms because similar biochemical and molecular processes occur in epidermal cells when fungal invasion occurs and during incompatible pollen tube penetration [13]. A large number of defense-related genes were speculated to be involved in incompatible pollen tube rejection [3, 32]. Ubiquitin mediated proteolysis pathway has a pivotal role in S-RNase-based GSI system. The Skp-1-Cullin1-F-box-Rbx1 complex (SCF) causes ubiquitin-mediated degradation of non-self S-RNase [33].

### Differentially expressed genes involve in SI

**Fig. 4:**
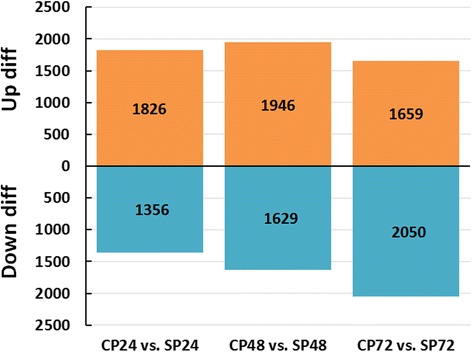
Number of differentially expressed unigenes between each two sample combination

To identify differentially expressed genes associated with SI in tea, the expression levels of unigenes were measured based on the fragments per kb per million of the mapped reads (FPKM) value. Totals of 3182, 3575 and 3709 differentially expressed genes were obtained between groups of CP24 vs. SP24, CP48 vs. SP48 and CP72 vs. SP72, respectively (Fig. 4, Additional file 3). To determine the putative functions of these genes, GO and KEGG analyses were implemented. Between the group comparing CP24 vs. SP24, 1826 genes were up-regulated and 1356 genes were down-regulated. Among these differentially expressed genes, 1008 were associated with GO categories, and 272 were mapped to 232 KEGG pathways. Similarly, between the group of CP48 vs. SP48, 1946 genes were up-regulated and 1629 genes were down-regulated; 1501 of them were associated with GO categories, and 566 were mapped to 251 KEGG pathways. In addition, between the group of CP72 vs. SP72, 1659 genes were up-regulated and 2050 genes were down-regulated; 1465 of them were associated with GO categories, and 485 were mapped to 266 KEGG pathways. The high number of differentially expressed unigenes provided an abundant list of candidate SI-related genes. In contrast, cDNA-AFLP analysis of self-incompatible lines of tea previously yielded only 100 polymorphic bands [26]. Therefore, RNA-seq seems to be more productive than cDNA-AFLP for identifying potential SI related genes.

In the above comparisons, some pathways occurred in all three comparisons, such as “phenylpropanoid biosynthesis”, “phenylalanine metabolism” and “plant-pathogen interaction”. In addition, “ubiquitin mediated proteolysis” [34–36], “apoptosis” [37, 38], “calcium signaling pathway” [30] and several defense-related pathways [3, 39] were also identified as up-regulated pathways that are associated with the SI reaction in plants. Therefore, the unigenes associated with the above pathways may also be associated with SI interaction in tea.

For “ubiquitin mediated proteolysis”, a SCF complex plays a key role in the S-RNase-based GSI system [1, 40]. S-phase kinase-associated protein 1 (Skp-1), F-box, RING-box protein 1 (Rbx1) and Cullin-1 (CUL1) also function as components of the SCF complex [33]. The SCF complex uniquely mediates the ubiquitination of non-self S-RNase, which is then degraded by the 26 s proteasome [33]. In this study, 7 differentially expressed unigenes annotated as *Skp-1* (CL1Contig5289 and CL18002Contig1), *F-box* (CL1Contig1120 and CL1Contig2140), *Rbx1* (CL1Contig679) and *CUL1* (CL1Contig1054 and comp136094_c0_seq6) were identified. These unigenes might be directly related to SI in tea, which warrant further exploration.

Calcium (Ca^2+^) is a second messenger, which plays a key role in pollen germination and tube growth and serves an important function in the prevention of self-fertilization [30]. Several Ca^2+^ signaling-related genes were identified that may function during SI. Calcium-dependent protein kinase (CDPK) is a Ca^2+^ response element, which localized at the tip of the pollen tube. In *Petunia*, overexpression the Pi *CDPK1* and the Pi *CDPK2* will disrupt growth polarity or inhibit extension of the pollen tube, respectively [41]. Five predicted *CDPKs* (CL1Contig1101, CL1Contig1911, comp91595_c0_seq1, CL41260Contig1 and comp128850_c0_seq1) were detected; the latter three have higher expression levels in SP samples than CP, which might affect the growth polarity or inhibit the extension of the pollen tubes from self-pollination. The protein families of calmodulin (CaM), calmodulin-like (CML), and calcineurin B-like (CBL) act as sensors during Ca^2+^ signal transduction. Several members of them are expressed pollen-specifically and highly expressed during pollen tube germination and elongation [42]. Here, three *CaMs* (CL20031Contig1, comp152366_c1_seq4 and CL2413Contig1), four *CBLs* (CL1Contig702, CL23964Contig1, CL26666Contig1 and comp88134_c0_seq1), and three *CMLs* (CL27939Contig1, CL33074Contig1, and CL39651Contig1) were identified to be differentially expressed. Therefore, the unigenes annotated as *CDPKs*, *CaMs* and *CMLs* merit special attention.

### Gene temporal expression patterns of CP and SP

**Fig. 5:**
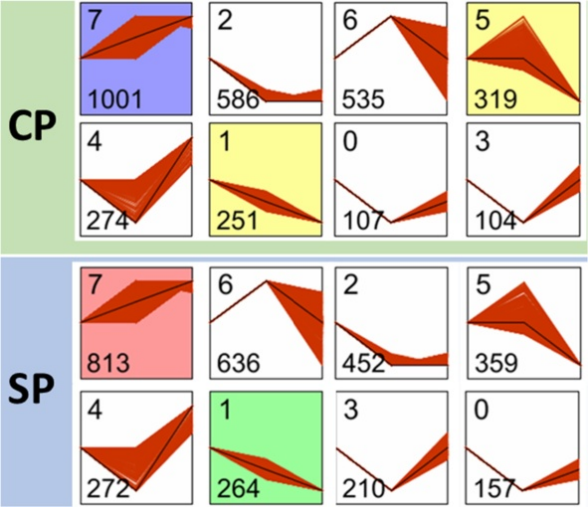
Different gene expression patterns between CP and SP. Each square represents a pattern. Top number indicates the profile ID number, bottom number indicates the number of time series genes. The patterns were ordered based on the number of genes. The colored squares were significant modules. The horizontal axis represents different times after pollination

To identify the temporal expression patterns of genes following self-pollination and cross-pollination treatments (Additional file 4), the short time-series expression miner software (STEM) was employed. The unigenes in CP and SP groups were clustered into 8 profiles (Fig. 5). Each profile represents a group of genes that exhibited similar expression trend. The highest number of genes in group CP was found in profile 7 (1,001, 31.51 %), followed by profile 2 (586, 18.45 %), profile 6 (535, 16.84 %) and finally profile 5 (319, 10.04 %). While in group SP, profile 7 (813, 25.70 %) was also the largest category, followed by profile 6 (636, 20.11 %), profile 2 (452, 14.29 %) and profile 5 (359, 11.35 %).

**Fig. 6:**
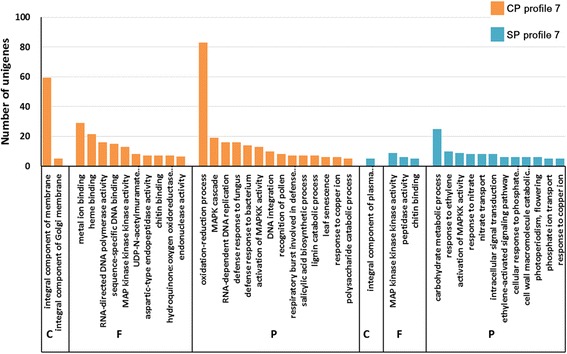
GO functional classification of the genes in profile 7. The letters C, F and P represent “Cellular component”, “Molecular function”, and “Biological process”, respectively

Following GO-term analysis to identify the putative functions of genes in profile 7 of both treatments, genes were classified into 3 categories including biological process, cellular component and molecular function (Fig. 6). For the biological process category, “oxidation-reduction process” was the most abundant subcategory in group CP, while 31 genes were classified into “defense response to fungus” (16), “recognition of pollen” (8) and “salicylic acid biosynthetic process” (7) categories. In group SP, “carbohydrate metabolic process” and “response to ethylene” were the dominant groups, while 12 genes were involved in “ethylene-activated signaling pathway” (6) and “cell wall macromolecule catabolic process” (6) subcategories. These results show that cross- and self-pollination result in different gene expression responses in the style.

**Fig. 7:**
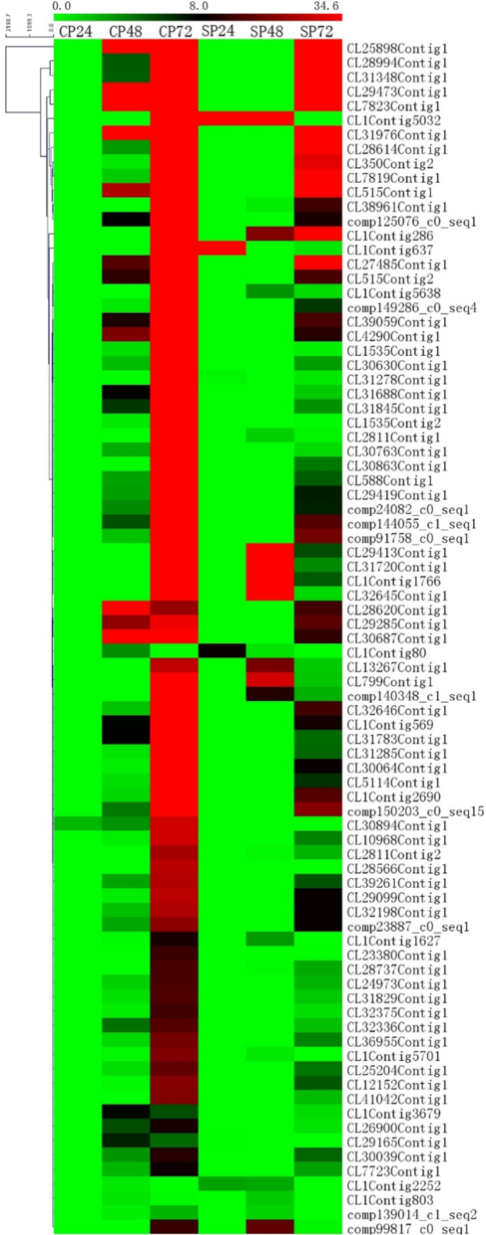
A heat map of 83 oxidation-reduction process-related unigenes generated using a MeV software. Along the horizontal axis (from left to right): CP24, CP48, CP72, SP24, SP48, SP72. The vertical axis represent the unigenes

**Fig. 8:**
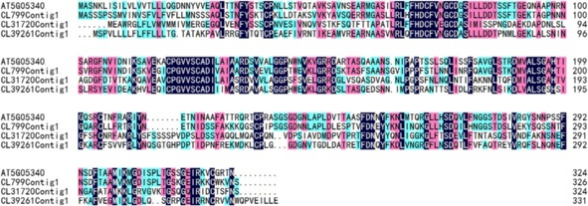
A multiple alignment of amino acid sequences of AT5G05340, CL799Contig1, CL39261Contig1 and CL31720Contig1 constructed by DNAMAN

A total of 83 unigenes were clustered into the “oxidation-reduction process” group, and a heat map was constructed based on the FPKM values of six samples (Fig. 7). Expression of these unigenes steadily increased from 24 h to 72 h in cross-pollinated styles. A similar trend was observed in self-pollinated styles but with a delay. Previous studies have revealed that reactive oxygen species (ROS) regulate stress resistance and pollen tube growth in plants [43, 44]. ROS concentrations increase in incompatible pollen tubes, leading to deleterious effects and SI-mediated PCD [45]. Various antioxidant systems take part in biological processes to maintain the proper concentration of ROS and prevent the damage from ROS [46]. Here, 6 peroxidases (CL39261Contig1, CL36955Contig1, CL799Contig1, CL31720Contig1, CL1Contig803 and CL13267Contig1), 1 polyphenol oxidase (CL28614Contig1) and several other redox-related genes were greatly increased in cross-pollinated styles, which is consistent with a previous study showing higher activity of peroxidase and polyphenol oxidase in cross-pollinated styles than self-pollinated ones of *C. sinensis* [47]. Among the 6 putative peroxidase genes, 3 unigenes (CL799Contig1, CL39261Contig1 and CL31720Contig1) contained intact ORFs and were homologues to AT5G05340. A multiple sequence alignment of AT5G05340 and the deduced amino acid sequences of CL799Contig1, CL39261Contig1 and CL31720Contig1 were constructed, which share approximately 70 %, 45 % and 41 % identity, respectively (Fig. 8). The gene AT5G05340 encodes a peroxidase (AtPrx 53) enzyme, which is closely related to the cell wall synthesis [48]. Therefore, the higher expression levels of these genes might promote pollen tube elongation during compatible styles.

Furthermore, 7 unigenes (CL1Contig2132, CL1Contig5305, CL20835Contig1, CL25392Contig1, CL29231Contig1, CL35779Contig1 and CL38632Contig1) associated with “salicylic acid biosynthetic process” were also consistently up-regulated in 3 samples of CP. Salicylic acid (SA) may contribute to regulating ROS accumulation [49], and then induce the defense responses and impact the self/non-self recognition as well as the pollen tube growth [50, 51].

### Differentially expressed gene validation by qRT-PCR

**Fig. 9:**
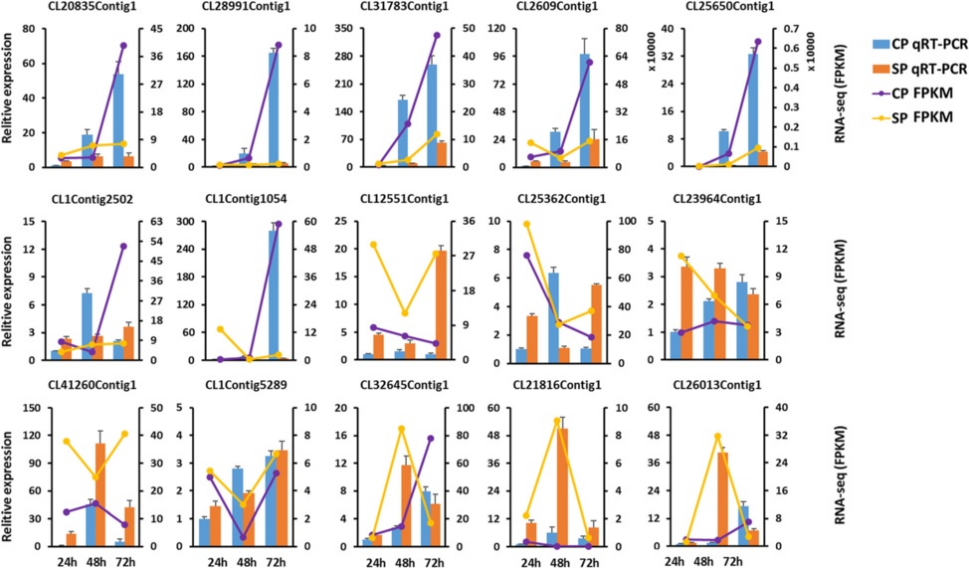
Validation of RNA-seq results by qRT-PCR, 15 genes were selected for qRT-PCR validation. The *GADPH* gene was chosen as the reference gene

Fifteen differentially expressed genes were selected to test the reliability of RNA-seq data (Table 3). These genes were associated with “plant-pathogen interaction” (CL1Contig2502, CL25362Contig1, CL25650Contig1, CL21816Contig1 and CL2609Contig1), “flavonoid biosynthesis” (CL32645Contig1, CL28991Contig1 and CL26013Contig1), “ubiquitin mediated proteolysis” (CL1Contig5289 and CL1Contig1054), “apoptosis” (CL12551Contig1), “calcium signaling pathway” (CL23964Contig1), “plant hormone response” (CL25362Contig1 and CL31783Contig1) and “negative regulation of programmed cell death” (CL20835Contig1). The expression patterns for 11 genes (73.3 %) were highly consistent with the sequencing data, while 4 genes (26.7 %, CL1Contig2502, CL25362Contig1, CL25362Contig1 and CL1Contig5289) were partially consistent (Fig. 9). The qRT-PCR results confirmed that the RNA-seq results were relatively accurate.

**Table 3:** Primers used for qRT-PCR validation

Unigenes	KEEG Annotation	Gene	Forward 5'–3'	Reverse 5'–3'
CL12551Contig1	Apoptosis	Serine/threonine-protein kinase	TATTCCCTTTTCCGATCTTCGCTAT	CAAAGATGAGTTGCTTTTCGTGTTG
CL23964Contig1	Calcium signaling pathway	calcineurin B-like protein	AAATTTGCATATCTGCCTGTGTCAA	ATGTAAAACATCCAAAACCCCAGTC
CL32645Contig1	Flavonoid biosynthesis	ANR	AGTATGGCTTTTGTGCTTTGATTGA	TAGAGGTGTCAAGGTTTCAGTTTCA
CL28991Contig1	Flavonoid biosynthesis	CYP75A(F3'5'H)	TTGTAAGTATGGGAGTTTGGGTAGG	CGACACATCATCAAGCGTAATAGAG
CL26013Contig1	Flavonoid biosynthesis	LDOX	AGTTGGCTAATAATGCTAGTGGTCA	TTTTCTAGTCGGCCTTCTTCTAGTC
CL20835Contig1	negative regulation of programmed cell death	Probable WRKY 40	ATTGTACTTGTCGCAAATGTCTGTT	CAATGTTAACCCACTTCCACTACAC
CL1Contig2502	Plant-pathogen interaction	caffeoyl-CoA O-methyltransferase	TCACAAGAACCAGACACAAACATTC	TTAATGGGCACAAGGGTTATGTTTC
CL41260Contig1	Plant-pathogen interaction	CPK	GATTCCTAAACATTTCCAAGCCACA	CTTTTAATGGTTGGACGGTGAGAAA
CL25650Contig1	Plant-pathogen interaction	PR1	ACCGTTATTTGTACACTGAACCCTA	TATGCTAATTCGAGGATGGGTGATT
CL21816Contig1	Plant-pathogen interaction	RPS2	CCAATGGGACCTCTGAACTATTTTG	TGCTTAAATTGCCTTTCAACGAAGA
CL2609Contig1	Plant-pathogen interaction	CALM	TGAAATCTATCATTCCATCCCCGTT	GAAGGGAGCTTTTGATGTGTTTGAT
CL1Contig5289	ubiquitin mediated proteolysis	Skp1	TGGGTTCCAATGGTATTGTTTGAAG	CGAGTGGATTATCGTTCAGTTGATG
CL1Contig1054	ubiquitin mediated proteolysis	Cullin-1	ATATGTGTGGTAACCCTATTGGTCC	AATTCCCCGTAAACCAGTTCATAGA
CL25362Contig1	Response to auxin	Protein TIFY 9	ATTGACGATTTTCTACAACGGAACC	TCTCCAGAAGTTGATGTTGATCTGT
CL31783Contig1	Response to salicylic acid	12-oxophytodienoate reductase 11	TCATAACAAGGGTGGGATCTTCTTT	CACTGTATCTTTCATGAACTGGTCG
CL25983Contig1	-	Ribonuclease, T2 family	CTGCGATAGCCGCAACTCTT	GGAAGTAGCTGTGCTGGTCAA
GE651107	Reference gene	GAPDH	GGAAGCATCATGAACTCAAAGTGAA	ATCCTTCTCATTGACACCCATAACA

### Analysis of a putative *S-RNase* gene

S-RNase is associated with GSI in plants of *Rosaceae*, *Solanaceae* and *Plantaginaceae* [8, 10]. Pistil-expressed S-RNase acts as a type of cytotoxin for self-pollen rejection [52]. It was first cloned from *Nicotiana alata* [51], then transgenic analyses in *Petunia* inflate prove that the S-RNase is the pistil determinant during the pollen rejection interaction [53]. Recently, S-RNase has been identified in various species, such as *Solanaceae* [51], *Prunus* [54], *Malus* [55], *Pyrus* [56], *Antirrhinum* [57], and *Citrus* [4].

**Fig. 10:**
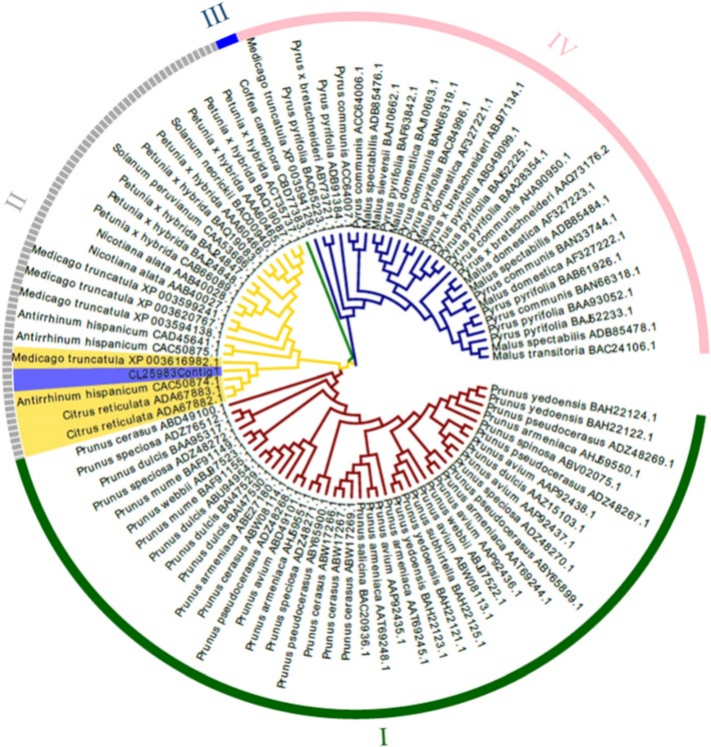
Phylogenetic analysis of the putative S-RNase (CL25983Contig1) from *C. sinensis* and S-RNases from other species using Neighbor-joining method. Blue highlighting indicates the position of CL25983Contig1. Yellow highlighting indicates the position of 4 genes, which have the highest similarity to CL25983Contig1

A putative *S-RNase* gene (CL25983Contig1), which had strong homology to *ribonuclease T2* gene, was obtained. A phylogenetic tree, based on the amino acid sequence of CL25983Contig1 and S-RNases from other species (Fig. 10), revealed 4 main groups. S-RNases from *Malus* and *Pyrus* were clustered in group I. The sequence from *Coffea canephora* was in group II. CL25983Contig1 and other S-RNases from *Petunia*, *Solanum*, *Nicotiana*, *Medicago*, *Antirrhinum* and *Citrus* were clustered in group III. All of the sequences from *Prunus* were clustered together in group IV. CL25983Contig1 was most closely related to S-RNases from *Citrus reticulata*, *Antirrhinum hispanicum* and *Medicago truncatulata*.

**Fig. 11:**
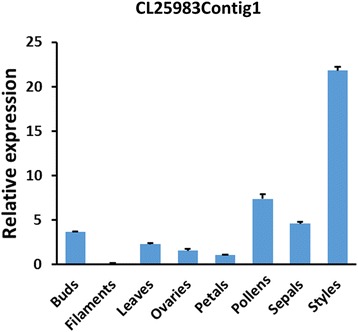
The expression pattern of the putative *S-RNase* gene (CL25983Contig1) in different tissues in *C. sinensis*

**Fig. 12:**
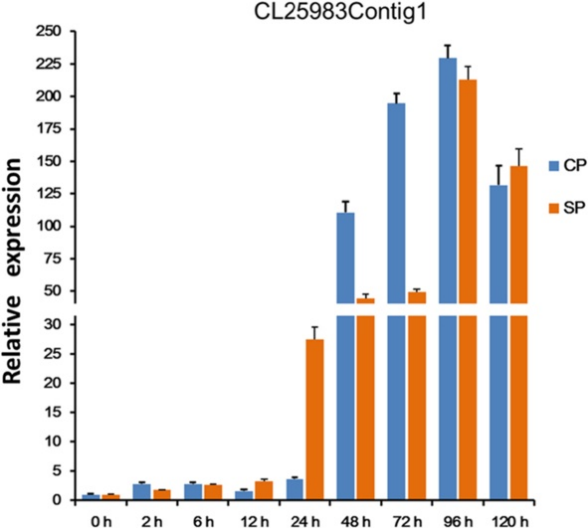
Expression pattern of the putative *S-RNase* gene (CL25983Contig1) after CP and SP

qRT-PCR of CL25983Contig1 in different tissues of tea (Fig. 11) confirmed that this gene was expressed at much higher levels in styles than other tissues such as filaments, leaves, petals and pollens. Moreover, the expression level of CL25983Contig1 increased at 24 h after self-pollination and was 7.45 times higher than that of cross-pollination (Fig. 12). Interestingly, the pollen tube growth in SP was hindered at 24 h. This suggests that CL25983Contig1 plays an important role in the SI reaction.

Recently, LSI has been demonstrated in many different species; however, the molecular mechanism for LSI is not well understood [6, 13]. LSI is defined based on the location of self-pollen inhibition rather than the molecular mechanism [12]. LSI can be regulated in different ways, through gametophytic or sporophytic control, either independently or acting together [13, 58]. For the pollen tube growth from a self-pollination that was arrested in the ovary, self-incompatibility in *C. sinensis* was assumed to occur via LSI [6, 23, 24]. Because growth of pollen tubes from self-pollination was halted in the style to a certain extent, and several SCF components-encoding genes and a putative *S-RNase* were identified, LSI in tea is likely under gametophytic control.

## Conclusions

Transcriptome analysis of styles after cross- and self-pollination in tea has identified a set of candidate genes involved in self-incompatibility. High levels of expression of several SCF components-encoding genes and a putative *S-RNase* gene suggest that LSI in *C. sinensis* might be under gametophytic control. Our study offers novel insights into the molecular mechanism behind SI in *C. sinensis*. We believe that this RNA-seq data will be useful for breeding and genomics research in *C. sinensis* as well as other plants in the Theaceae family.

## Methods

### Plant material

Two cultivars of ‘Fuding Dabaicha’ and ‘Zhongcha 108’, grown in the China National Germplasm Hangzhou Tea Repository, were used in this study. ‘Fuding Dabaicha’ is used in many tea-breeding programs in China and more than 18 % of national elite cultivars are derived from it [51]. While ‘Zhongcha 108’ is a radiation-induced cultivar selected by the Tea Research Institute Chinese Academy of Agricultural Sciences. The reproductive organs and seed-setting rate of these two cultivars are normal. The following tissues were collected from ‘Fuding Dabaicha’: buds, leaves, filaments, petals, pollens, sepals, unpollinated styles and ovaries with 3 biological replicates. The samples were frozen immediately in liquid nitrogen and stored at −80 °C.

### Pollination treatment

Pollens of ‘Fuding Dabaicha’ and ‘Zhongcha 108’ were periodically collected from “balloon” stage flowers by placing them in a desiccator at room temperature for 8 h. Pollination was performed from 9:00-11:00 am and 1:00-3:00 pm on sunny days in November. Pollination combinations included self-pollination (SP) of ‘Fuding Dabaicha’ × ‘Fuding Dabaicha’ and cross-pollination (CP) of ‘Fuding Dabaicha’ × ‘Zhongcha 108’. Flowers in the “balloon” stage were bagged after emasculation and self- or cross-pollinated. The styles were harvested at different intervals (0 h, 2 h, 6 h, 12 h, 24 h, 48 h, 72 h, 96 h and 120 h) after pollination. Therefore, the number of self-pollinated samples ranged from SP0 (0 h after pollination) to SP120 (120 h after pollination). Similarly, the number of cross-pollinated samples ranged from CP0 to CP120. After harvesting, half of the styles of each sample were fixed in FAA (formalin:acetic acid:alcohol 5:5:90 v/v) for 3 h. The remaining styles were divided into two parts, one for RNA-seq and another for qRT-PCR with three biological replicates. Finally, the materials were frozen in liquid nitrogen and stored at −80 °C.

### Observation of pollen tube growth

After removing the styles from FAA, the styles were macerated with 3 M NaOH for 3 h. Then, the styles were split along the vertical axis into 3 sections, and stained by 1 % aniline blue for 3 h. Finally, the pollen tubes were observed using a Nikon Eclipse 80i fluorescence microscope (Nikon Instech Co. Ltd., Kawasaki, Japan).

### RNA extraction, library construction and sequencing

Total RNAs were extracted from self- and cross-pollinated styles using an RNAprep pure Plant Kit (Tiangen, Beijing, China). The quality of the total RNA was confirmed by electrophoresis through a 1 % agarose gel followed by viewing, and using a NanoDrop 2000 (Thermo Scientific, DE, USA). RNAs from 6 samples (SP24, SP48, SP72, CP24, CP48, and CP72) were used for RNA-seq. The mRNA was isolated from 6 μg total RNA using the Truseq RNASample Prep Kit (Illumina, San Diego, CA, USA). Next, the mRNA was mixed with fragmentation buffer for mRNA fragmentation. After the short fragment, mRNAs were used as template for the first-strand cDNA synthesis using random hexamer-primers, the second-strand cDNA was synthesized and then purified using a SuperScript Double-Stranded cDNA Synthesis Kit (Invitrogen, Carlsbad, CA, USA) and PCR extraction kit (Takara Bio, Otsu, Japan), respectively. The cDNA library was then created following end-repair, polyA addition, adaptor ligation and PCR amplification. After that, cDNA libraries were evaluated using an Agilent 2100 Bioanalyzer (Agilent Technologies, Palo Alto, Calif.) and sequenced on an Illumina HiSeq™ 2000.

### *De novo* assembly and annotation

Raw RNA-seq data were preprocessed by first removing adaptor and low quality sequences (Q < 20 or less than 35 bp) and the qualified reads were then assembled into non-redundant transcripts by Trinity [59] and TGICL [60]. All non-redundant transcripts were annotated to the NR, SWISSPROT, KOG and KEGG databases by blastx using a threshold of 1e^−5^. GO term distribution for each transcript was carried out using Blast2GO software [61].

### Differential gene expression analysis

The expression of each gene was estimated by FPKM, which was calculated based on the universal reads from 6 separate libraries [62]. Seven groups including CP24 vs. CP48, CP48 vs. CP72, CP24 vs. SP24, CP48 vs. SP48, CP72 vs. SP72, SP24 vs. SP48 and SP48 vs. SP72 were compared. To identify differentially expressed genes, differential expression analysis of the comparisons was performed using a DESeq program (http://bioconductor.org/packages/release/bioc/html/DESeq.html). *P*-values related to the analysis of differentially expressed genes were adjusted for multiple testing by the Benjamini-Hochberg false discovery rate (FDR) method [63]. For each comparison, we extracted those unigenes with a criteria of FDR-adjusted *P*-value ≤ 0.05 and |log2Ratio| > 1. Furthermore, the differentially expressed unigenes were annotated to the KEGG database using blastx. GO term distribution was performed by a Blast2go software using an FDR-adjusted *P*-value ≤ 0.05 as the cutoff. The genes, which expressed differentially in the comparison of CP24 vs. SP24, CP48 vs. SP48 and CP72 vs. SP72, were used to screen candidate genes for SI.

### Comparison of differential gene expression patterns between CP and SP

A STEM software (Carnegie Mellon University, USA) was employed to analyze different patterns of gene expression between CP and SP. Differentially expressed genes in the comparisons of CP24 vs. CP48 and CP48 vs. CP72 were used to cluster changing patterns of CP treatments (Additional file 3). Similarly, differentially expressed genes in the comparison of SP24 vs. SP48 and SP48 vs. SP72 were used to cluster changing patterns of SP treatments (Additional file 3). GO-term analysis was utilized to determine the potential functions of times series genes. GO term with a FDR-adjusted *P*-values ≤ 0.05 was considered to be significantly enriched. The heat map was generated using MeV software (www.tm4.org/mev.html). The multiple alignments of amino acid sequences were constructed using DNAMAN software (www.lynnon.com).

### Validation by quantitative real-time PCR (qRT-PCR)

Fifteen differentially expressed transcripts were selected for validation of sequencing data using qRT-PCR. Specific primers were designed by Primer 5 software (Premier Biosoft International, Palo Alto, CA). Total RNA was extracted using an RNAprep pure Plant Kit (Tiangen, Beijing, China) from the styles. One microgram of total RNA was used to synthesize cDNA using a PrimeScript® 1st Strand cDNA Synthesis Kit (Takara, Dalian, China). Then, qRT-PCR was performed on the ABI 7500 Real-Time PCR System (Applied Biosystems) using a PrimeScript™ RT reagent qPCR Kit (Takara, Dalian, China). A *GADPH* (GE651107) gene was utilized as the reference gene. The real time PCR program was as follows: 95 °C for 15 s, 40 cycles at 95 °C for 5 s, 60 °C for 34 s. Each reaction was repeated three times for biological and technical replicates, respectively. The relative quantitation of the gene expression was calculated using the 2^−ΔΔCt^ method [64].

### Expression analysis of a putative *S-RNase* gene

The amino acid sequence of CL25983Contig1 was predicted using a DNAstar program Editseq option (DNASTAR, Inc., Madison, Wis.). A phylogenetic tree was constructed using the amino acid sequence deduced from CL25983Contig1 and 93 S-RNases from other species by a Neighbor-joining method by using MEGA 5.2 (www.megasoftware.net). Total RNA was extracted from 8 different tissues. The expression pattern of CL25983Contig1 was examined using qRT-PCR with a reference gene *GAPDH*. Furthermore, the expression profiling of CL25983Contig1 was detected in both two pollination treatments from 0 h to 120 h after pollination. Each reaction was performed with three biological replicates and three technical replicates.

### Availability of supporting data

Sequence data of 6 samples have been deposited in the NCBI SRA database (SRR3290042, SRR3290055, SRR3290062, SRR3290078, SRR3290084 and SRR3290124).

## Additional files

Additional file 1: The species distribution of all of the unigenes. (TIF 3926 kb) Additional file 2: Gene Ontology terms of all of the unigenes. (TIF 9114 kb) Additional file 3: Differentially expressed genes between cross- and self-pollination samples. (XLSX 1339 kb) Additional file 4: Temporal expression of genes in cross- and self-pollination samples. (XLSX 1737 kb)

## Abbreviations

SP: self-pollination
CP: cross-pollination
FPKM: fragments per kb per million mapped reads
SSI: sporophytic self-incompatibility
SRK: S-receptor kinase
SCR: S-locus cysteine-rich protein
GSI: gametophytic self-incompatibility
SLF/SFB: S-locus F-box protein
PCD: programmed cell death
OSI: ovarian self-incompatibility
LSI: late-acting self-incompatibility
NGS: next generation sequencing
*C. sinensis*: *Camellia sinensis*
PR-1: pathogenesis-related-1 protein
NAA: 1-naphthaleneacetic acid
SCF: Skp-1-Cullin1-F-box-Rbx1 complex
Skp-1: S-phase kinase-associated protein 1
Rbx1: RING-box protein 1
CUL1: Cullin-1
CDPK: calcium-dependent protein kinase
CaM: calmodulin protein
CML: calmodulin-like protein
CBL: calcineurin B-like protein
STEM: short time-series expression miner software
ROS: reactive oxygen species
SA: salicylic acid
qRT-PCR: Quantitative real-time PCR
KOG: clusters of orthologous groups for eukaryotic complete genomes
NR: NCBI non-redundant protein
KOG: clusters of orthologous groups for eukaryotic complete genomes
KEGG: Kyoto encyclopedia of genes and genomes
Swiss-Port: Swiss-Prot protein database
GO: gene ontology
NCBI: National Center for Biotechnology Information
RNA-Seq: RNA sequencing
FDR: false-discovery rate
