# Supplementary figures and images for: Transcriptome analysis reveals self-incompatibility in the tea plant (Camellia sinensis) might be under gametophytic control
Source: BMC Genomics. 2016 May 17;17:359. doi: 10.1186/s12864-016-2703-5 (PMC4869358; doi:10.1186/s12864-016-2703-5)

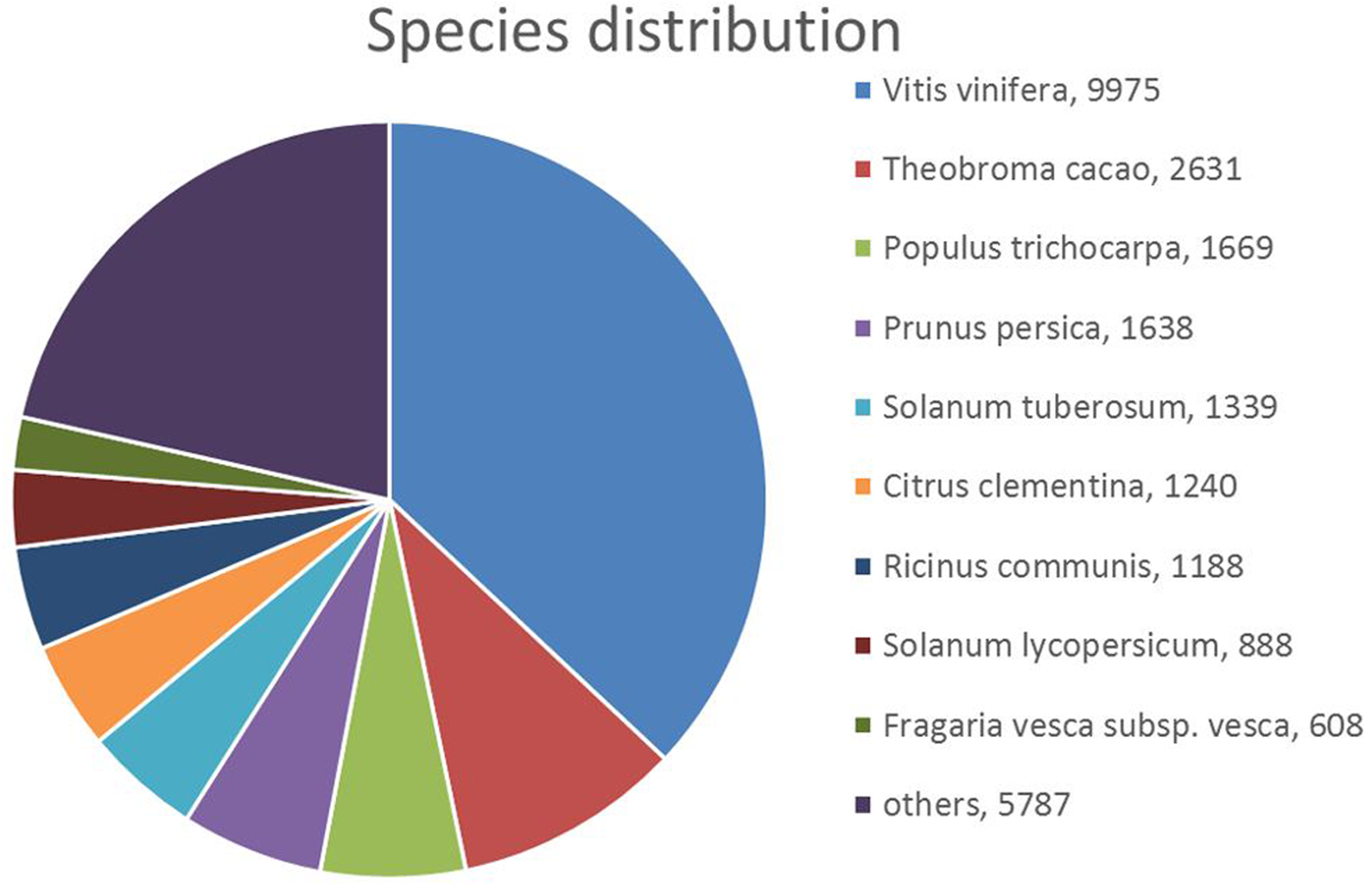

Supplement: Additional file 1: — The species distribution of all of the unigenes. (TIF 3926 kb) [file 12864_2016_2703_MOESM1_ESM.tif]

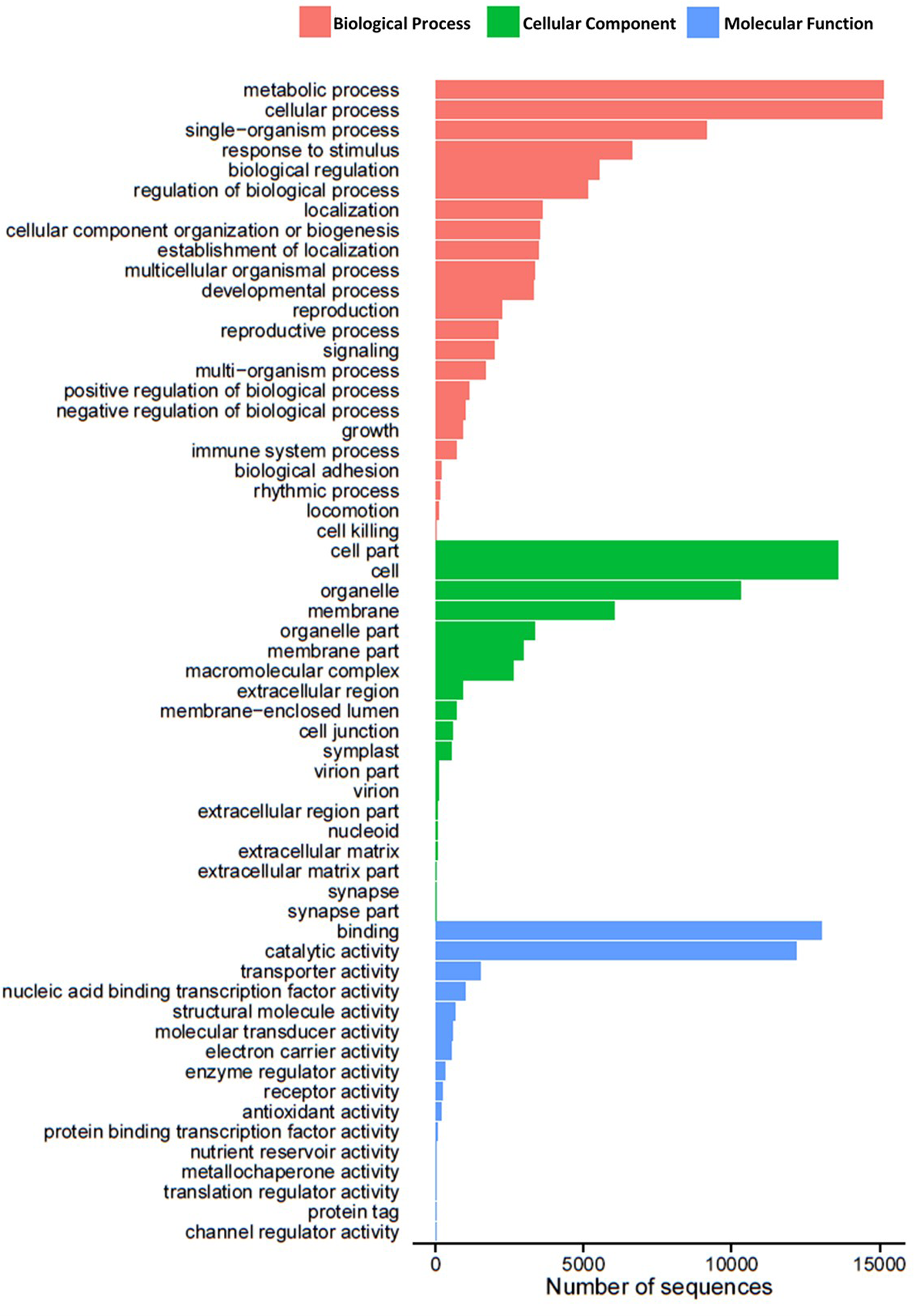

Supplement: Additional file 2: — Gene Ontology terms of all of the unigenes. (TIF 9114 kb) [file 12864_2016_2703_MOESM2_ESM.tif]
